# AI Lung Segmentation and Perfusion Analysis of Dual-Energy CT Can Help to Distinguish COVID-19 Infiltrates from Visually Similar Immunotherapy-Related Pneumonitis Findings and Can Optimize Radiological Workflows

**DOI:** 10.3390/tomography8010003

**Published:** 2021-12-23

**Authors:** Andreas S. Brendlin, Markus Mader, Sebastian Faby, Bernhard Schmidt, Ahmed E. Othman, Sebastian Gassenmaier, Konstantin Nikolaou, Saif Afat

**Affiliations:** 1 Department of Diagnostic and Interventional Radiology, Eberhard-Karls University, D-72076 Tubingen, Germany; Andreas.Brendlin@med.uni-tuebingen.de (A.S.B.); Markus.Mader@med.uni-tuebingen.de (M.M.); Ahmed.E.Othman@googlemail.com (A.E.O.); Konstantin.Nikolaou@med.uni-tuebingen.de (K.N.); Saif.Afat@med.uni-tuebingen.de (S.A.); 2 Siemens Healthcare GmbH, Computed Tomography, D-91301 Forchheim, Germany; Sebastian.Faby@siemens-healthineers.com (S.F.); Bernhard.Schmidt@siemens-healthineers.com (B.S.); 3 Department of Neuroradiology, University Medical Center, D-55131 Mainz, Germany

**Keywords:** COVID-19, dual energy, tomography, X-ray computed, artificial intelligence

## Abstract

(1) To explore the potential impact of an AI dual-energy CT (DECT) prototype on decision making and workflows by investigating its capabilities to differentiate COVID-19 from immunotherapy-related pneumonitis. (2) Methods: From 3 April 2020 to 12 February 2021, DECT from biometrically matching patients with COVID-19, pneumonitis, and inconspicuous findings were selected from our clinical routine. Three blinded readers independently scored each pulmonary lobe analogous to CO-RADS. Inter-rater agreement was determined with an intraclass correlation coefficient (ICC). Averaged perfusion metrics per lobe (iodine uptake in mg, volume without vessels in ml, iodine concentration in mg/mL) were extracted using manual segmentation and an AI DECT prototype. A generalized linear mixed model was used to investigate metric validity and potential distinctions at equal CO-RADS scores. Multinomial regression measured the contribution “Reader”, “CO-RADS score”, and “perfusion metrics” to diagnosis. The time to diagnosis was measured for manual vs. AI segmentation. (3) Results: We included 105 patients (62 ± 13 years, mean BMI 27 ± 2). There were no significant differences between manually and AI-extracted perfusion metrics (*p* = 0.999). Regardless of the CO-RADS score, iodine uptake and concentration per lobe were significantly higher in COVID-19 than in pneumonitis (*p* < 0.001). In regression, iodine uptake had a greater contribution to diagnosis than CO-RADS scoring (Odds Ratio (OR) = 1.82 [95%CI 1.10–2.99] vs. OR = 0.20 [95%CI 0.14–0.29]). The AI prototype extracted the relevant perfusion metrics significantly faster than radiologists (10 ± 1 vs. 15 ± 2 min, *p* < 0.001). (4) Conclusions: The investigated AI prototype positively impacts decision making and workflows by extracting perfusion metrics that differentiate COVID-19 from visually similar pneumonitis significantly faster than radiologists.

## 1. Introduction

COVID-19, the disease caused by severe acute respiratory syndrome coronavirus 2 (SARS-CoV-2), was declared a global emergency by the WHO in January 2020 [1]. More than 20 months after the initial outbreak, COVID-19 is still one of the major healthcare burdens worldwide, with over 254,492,345 confirmed cases and a global death toll of over 5,117,529 [2]. Computed tomography (CT) has always played an essential role in this pandemic, not only for diagnosis but also for follow-up after the acute phase of the disease [3,4]. Ground-glass opacities, consolidations, and septal thickenings have been described as typical findings for COVID-19 [5]. The COVID-19 Reporting and Data System (CO-RADS), an established assessment scheme based on evaluating these findings, is reported to have a substantial interobserver agreement in the categories of the highest and lowest likelihood for the presence of COVID-19 [6]. As the above-described findings are rather unspecific for COVID-19 pneumonia, differentiating COVID-19 from other diseases with a comparable visual impression may prove challenging in unclear cases [7]. Other inflammatory pulmonary diseases like immunotherapy-related pneumonitis may mimic the visual impressions of COVID-19 [8]. For patients undergoing immunotherapy, distinguishing these two entities is time-critical, as both require fast but significantly different therapeutic approaches [9,10]. Even outside this setting, though, false-negative reverse transcription polymerase chain reaction (rt-PCR) tests may delay proper care and even put other patients at risk of infection [11]. Besides the established scoring systems, prominent methods to facilitate COVID-specific diagnoses based on radiological imaging have included artificial intelligence (AI) tools [12,13]. In medical imaging, convolutional neural networks (CNN) have shown great potential to facilitate radiological workflows due to their high classification capabilities [14]. Furthermore, dual-energy CT (DECT) has been shown to outperform the diagnostic capabilities of single-energy CT because of its superior exploitation of spectral information and its inherent material decomposition capabilities [15]. Via DECT-generated iodine quantification maps, it was previously shown that COVID-19 is associated with pulmonary perfusion disorders [16]. Therefore, we aimed to combine these approaches and investigate the performance of an AI-based DECT lung perfusion analysis in differentiating COVID-19 findings from immunotherapy-related pneumonitis [17]. We hypothesize that the AI prototype extracts valid perfusion metrics, that DECT perfusion metric analysis can help differentiate entities, and that implementation of the AI prototype may be beneficial to radiological workflows.

## 2. Materials and Methods

### 2.1. Study Design and Population

The institutional review board approved retrospective image data collection for this single-center study’s purpose with a waiver for the need for informed consent (609/202BO). From 3 April 2020 to 12 February 2021, whole-body DECT to rule out foci of infection from patients with symptomatic COVID-19, melanoma patients with symptomatic immunotherapy-related pneumonitis (checkpoint inhibitors: anti-CTLA-4, anti-PD1, or a combination of both), and patients with inconspicuous pulmonary findings were selected from our clinical routine. We collected the patients’ age, sex, height, and weight. The patients’ BMI was computed, as obesity is a known risk factor in patients with COVID-19 [18]. As inclusion criteria for further analyses, we chose non-intubated patients from all three groups with exactly matching biometric profiles (same age, same sex, same BMI). As exclusion criteria, we chose factors that might limit pulmonary perfusion other than infection (e.g., heart failure, pulmonary effusion, embolisms, malignant lesions). If COVID-19 and pneumonitis patients had more than one examination in the given timeframe, we selected the examination closest to the clinical diagnosis.

### 2.2. Image Acquisition and Reconstruction Parameters

All DECT were contrast-enhanced (Imeron 400, Bracco, Milan, Italy) whole-body examinations and performed on the same 3rd generation dual-source CT scanner (SOMATOM Force; Siemens Healthineers, Erlangen, Germany). Contrast agent (patients’ bodyweight in kg + 15 = contrast agent in mL) as well as a subsequent saline flush (40 mL) were administered through a peripheral vein cannula by a double syringe power injector (Medrad; Bayer, Leverkusen, Germany) at a flow rate of 2.5 mL/s. Image acquisition took place in a portal venous phase (90 s after the application). Attenuation-based tube current modulation (CARE Dose4D, reference mAs 190) was activated for the examination. Tube voltage was set to 100/Sn150 (tube A 100 kV, tube B tin-filtered 150 kV). Collimation was set to 0.6 × 192/128 mm, pitch was 0.6, and gantry rotation time 0.5 s. A quantitative medium-soft kernel without overshoots (Qr40d) was used with iterative beam hardening correction (IBHC) set to iodine for image reconstruction. The CT datasets were reconstructed in axial orientation with a slice thickness and an increment of 1 mm.

### 2.3. Subjective Reading and CO-RADS Scoring

The datasets were anonymized and randomized by a member of our group, who was not associated with subjective reading. Three fully trained radiologists with experience ranging from 1 to 8 years independently performed readings and CO-RADS scoring [6]. We expected perfusion metrics to differ due to the Euler-Liljestrand effect. Therefore, we decided to additionally score each side and each pulmonary lobe individually, resulting in a total of 840 (3 × 35 × 8) scores per reader.

### 2.4. Lung Segmentation and Perfusion Analysis

We measured mean iodine uptake in mg, mean lung volume (without vessels) in ml, and mean iodine concentration in mg/mL for each pulmonary lobe individually, the whole left/right side, and the whole lung. These DECT metrics were acquired for each patient by manual segmentation using syngo.CT DE Lung Analysis (syngo.via VB40, Siemens Healthineers, Erlangen, Germany) performed by our senior radiologist who is proficient with the software. Subsequently, each patient was again analyzed employing a previously described AI prototype that applies a convolutional neural network to the DECT volumes for fissure segmentation and the automated extraction of quantitative perfusion metrics using binary lung lobe masks (eXamine DE Lung Isolation prototype, Siemens Healthineers, Erlangen, Germany) [19]. The time to diagnosis (until the DECT metric extraction was finished) was measured for both methods.

### 2.5. Statistical Analysis

Figures and Graphs were created using GraphPad Prism version 9.0.2 for Windows (GraphPad Software, San Diego, CA, USA). We used IBM^®^ SPSS^®^ Statistics Version 27 for Windows (Armonk, NY, USA) for the statistical analysis of patient data. Data distribution was tested using the Shapiro–Wilk test. Normally distributed variables were expressed as mean ± standard deviation and non-normally distributed variables as median and interquartile range (IQR). Data analysis ensued using a generalized linear mixed model (GLMM). The Greenhouse–Geisser correction was used in case of violation of sphericity. The Bonferroni correction was used for multiple comparisons to counteract Type 1 error increase. A *p*-value ≤ 0.05 indicated statistical significance. As we limited patient inclusion to biometrically matched pairs in three groups, a post hoc sensitivity analysis was added using G*Power (ver. 3.1.9.7) to quantify the minimal detectable effect size in our setup [20,21]. To measure the inter-rater agreement of CO-RADS scores, we used an intraclass correlation coefficient (ICC, absolute agreement, average measures) [22]. ICC values of 0–0.2 were considered as slight, 0.21–0.4 as fair, 0.41–0.6 as moderate, 0.61–0.8 as substantial, and 0.81–1.00 as almost perfect levels of agreement. A multinomial regression analysis [23,24] was utilized to investigate the contribution of the items “Reader” (R1, R2, R3), “CO-RADS Score” (CO-RADS 1,2,3,4,5), and three DECT metrics: “iodine uptake” (mean ± SD per pulmonary lobe), “volume (without vessels)” (mean ± SD per pulmonary lobe), and “iodine concentration” (mean ± SD per pulmonary lobe) to the differentiation of inconspicuous findings from COVID-19 and from immunotherapy-related pneumonitis. Goodness-of-fit was tested using a χ^2^ likelihood-ratio test (LRT), Pearson χ^2^, and Nagelkerke Pseudo-R^2^.

## 3. Results

### 3.1. Study Population and CO-RADS Score

**Figure 1:**
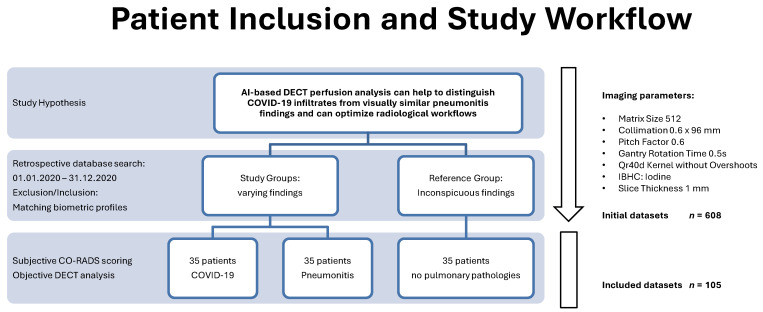
Patient inclusion and study workflow.

The initial database search revealed 75 patients with COVID-19, 138 patients with immunotherapy-related pneumonitis, and 395 patients without pulmonary pathologies. From these, we excluded 503 patients not meeting our inclusion criteria and selected a total of 105 patients (35 patients for each subgroup) for further analyses. With an α of 0.05 and a power (1-β) of 0.95, power analysis showed the minimal detectable effect size for significant differences in our setup (105 patients, 3 matched groups, 24 repeated measures) to be small (f = 0.11), verifying the validity of our results. Figure 1 illustrates patient inclusion and the study workflow. Table 1 gives an overview of the patient characteristics in the respective subgroups.

The inter-rater agreement for lobe-wise CO-RADS scoring was almost perfect (ICC = 0.86; *p* ≤ 0.001). The number of pulmonary lobes classified in the specific CO-RADS score groups is shown in Table 2.

### 3.2. Dual-Energy CT Metric Comparison

#### 3.2.1. Method Validation and Time to Diagnosis

**Figure 2:**
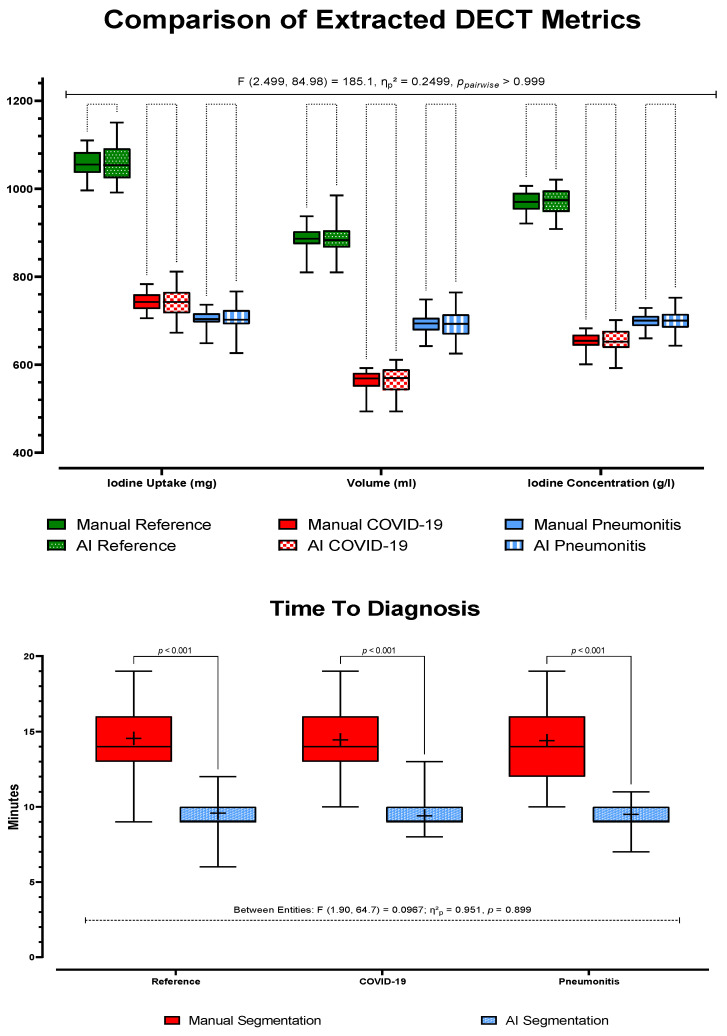
Comparison of extracted DECT metrics and time to diagnosis.

There were no significant differences (*p* > 0.999) in pairwise comparisons between the DECT metrics extracted by manual segmentation and the metrics automatically extracted by the AI prototype. However, at 10 ± 1 min, the time to diagnosis was significantly shorter when using the AI prototype than at 15 ± 2 min when using manual segmentation (*p* < 0.001). See Figure 2 for further details.

#### 3.2.2. Analysis of AI-Based Lung Segmentation

**Figure 3:**
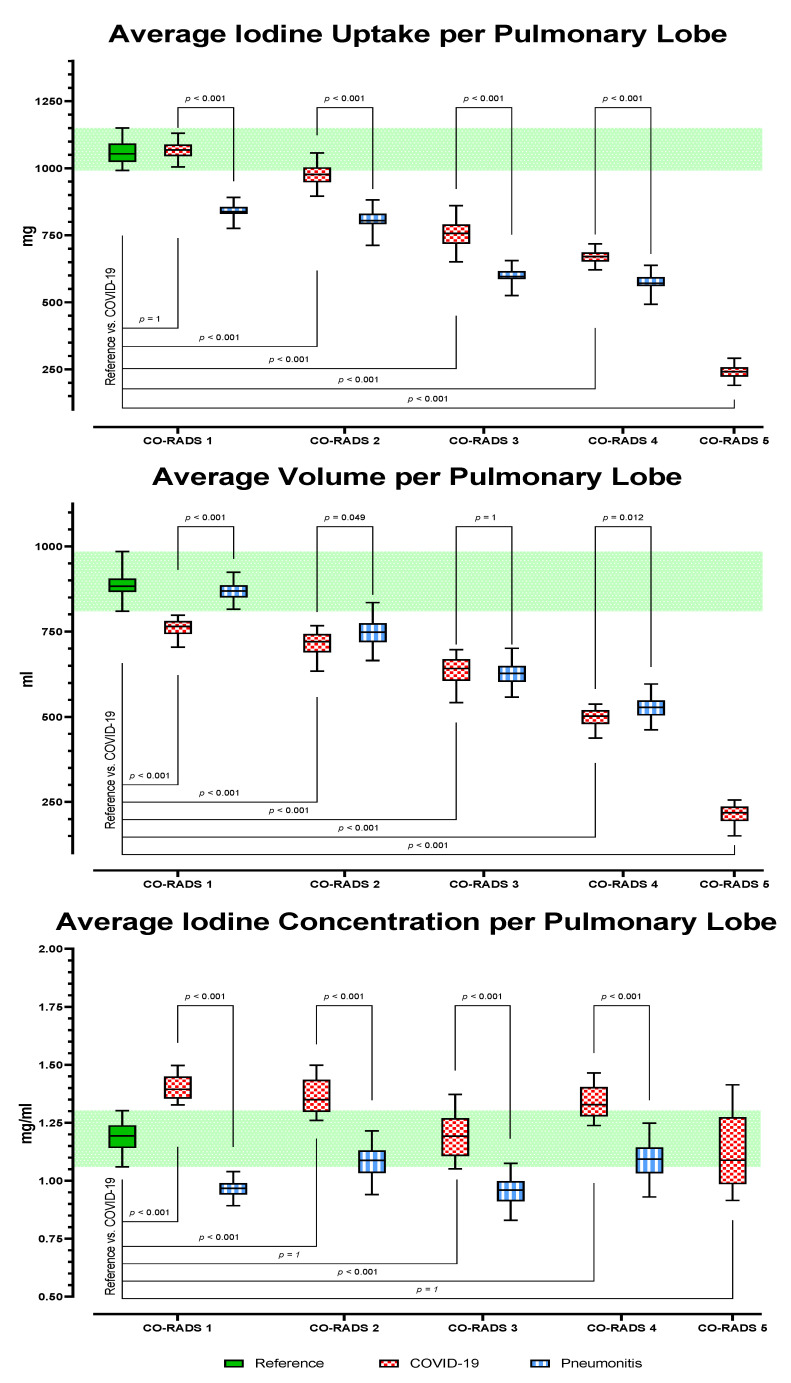
Averaged DECT metrics per Pulmonary Lobe.

**Figure 4:**
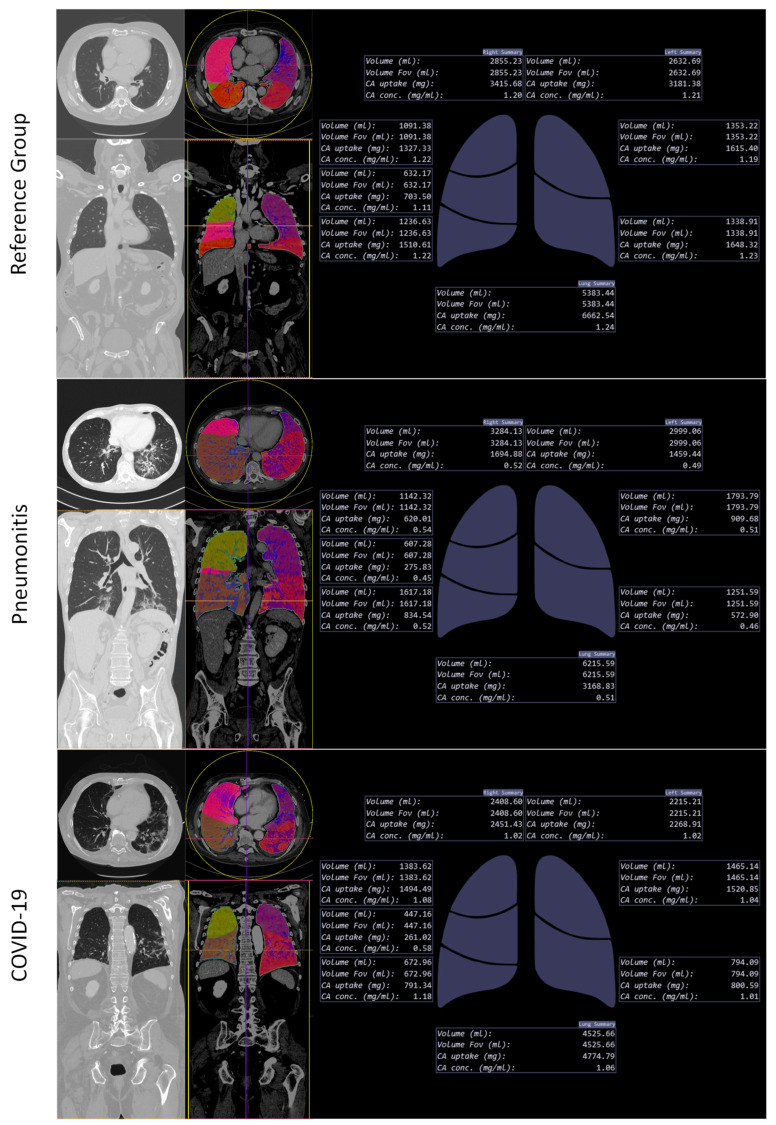
AI DECT lung segmentation and analysis in three patients (Reference, Pneumonitis, and COVID-19 with similar findings).

For each item (iodine uptake, volume, and iodine concentration per pulmonary lobe), GLMM showed significant variance between the subgroups (F (1258, 4279) = 558.0, η_p_^2^ = 0.419, *p* ≤ 0.001). At equal visual CO-RADS score levels, post hoc tests showed COVID-19 to have a significantly higher iodine uptake per pulmonary lobe and a significantly higher iodine concentration per pulmonary lobe than pneumonitis (*p* < 0.001). See Figure 3 for further details about average iodine uptake, average volume, and average iodine concentration per pulmonary lobe. Of special interest are the subanalyses of CO-RADS scores 2–4, where clinical routine shows substantial visual overlap. Figure 4 is an example of AI DECT lung segmentation and perfusion analysis in three patients.

In multinomial regression, statistical analysis showed a good model fit (LRT: χ^2^ (10) ≥ 568.34, *p* ≤ 0.001; Pearson χ^2^ (624) ≥ 1307.81, *p* ≥ 0.275; Nagelkerke Pseudo-R^2^ ≥ 0.67). The combination of the items “Reader” (R1, R2, R3), “CO-RADS Score” (CO-RADS 1,2,3,4,5), “Iodine Uptake”, “Volume”, and “Iodine Concentration” accounted for a significant amount of variance in the outcome (*p* < 0.001). The item “Reader” did not contribute to the model (*p* = 0.109). CO-RADS scores were a significant contributor towards infiltrate differentiation (B = −0.88, std. error (SE) = 0.02, *p* ≤ 0.001). However, pulmonary lobes with COVID-19 only had a slightly elevated likelihood of having higher CO-RADS scores (Odds Ratio (Exp. B) = 0.92 [95%CI 0.88–0.95]) than pulmonary lobes with pneumonitis. The DECT metric “Iodine Uptake” was not only a significant contributor towards differentiating infiltrates (B = 0.6, std. error (SE) = 0.25, *p* = 0.019), the likelihood of correct classification by iodine uptake was higher (Odds Ratio (Exp. B) = 1.82 [95%CI 1.10–2.99]) than by visual classification (Odds Ratio (Exp. B) = 0.20 [95%CI 0.14–0.29]). See Table 3 for further details.

## 4. Discussion

The purpose of this study was a threefold evaluation of an AI-based dual-energy CT lung segmentation and analysis prototype: First, to validate the perfusion metrics extracted by the prototype. Second, to investigate the capabilities of automatically extracted perfusion metrics to differentiate COVID-19 infiltrates from visually similar immunotherapy-related pneumonitis findings and compare the metrics’ relative significance to that of visual CO-RADS scoring. Third, to analyze potential benefits which the implementation of the prototype has on radiological workflows. For this purpose, we retrospectively compared DECT datasets of 35 patients with proven and symptomatic COVID-19 infection to visually similar findings of 35 patients with proven and symptomatic pneumonitis. In addition, as a reference group, we included 35 patients with matching biometric profiles who had no pulmonary pathologies. DECT analysis showed perfusion metrics with a high discriminatory power between COVID-19 and visually similar pneumonitis findings, emphasizing the capabilities of DECT in visualizing biological and pathophysiological processes. Furthermore, we found no differences between the DECT perfusion metrics extracted by manual segmentation and those extracted by the AI prototype. However, the time to diagnosis was significantly shorter when using the AI prototype than when using manual segmentation. CO-RADS is a categorial CT assessment scheme to evaluate the likelihood of the presence of COVID-19. As discussed by Prokop et al., the scores for the almost certain absence/presence of COVID-19 had a substantial inter-reader agreement [6]. In unclear or rather unlikely cases however, they reported only moderate agreement levels. Bai et al. further pointed out the low specificity of the typical COVID-19 findings in chest CT, especially in synopsis with the low sensitivity of reverse-transcription polymerase chain reaction (rt-PCR) testing in earlier disease stages [25]. Our data is concordant to the results of these two studies, as COVID-19 patients had only a slightly elevated likelihood of having higher CO-RADS scores than patients with pneumonitis. Since there was no significant contribution in CO-RADS by single readers to determinate disease entity, and scores were given with almost perfect agreement levels, we need to reiterate the need for more sophisticated diagnosis methods than visual assessment alone. Other studies have investigated the role of DECT lung perfusion analysis in patients with COVID-19. Oudkerk et al. showed severely impaired lung perfusion in COVID-19 patients with thromboembolic complications [26]. Perfusion deficits in patients with COVID-19 are, however, described even without any complications whatsoever. Grillet et al. reported lower pulmonary iodine levels even in visually inconspicuous parenchyma, giving evidence for microvascular disease [27,28]. The damaging effect of SARS-CoV-2 spike proteins on endothelial function is confirmed in newer studies [29]. Concordantly, patients with COVID-19 generally had significantly higher pulmonary iodine uptake at lower average lobe volumes than patients with pneumonitis and patients without pulmonary pathologies. Lang et al. described mosaic perfusions in patients with COVID-19 that were unlikely caused by airway disease [30]. Afat et al. reiterated these findings and described mismatches of perfusion deficits and ground-glass opacities in patients with COVID-19 [16]. We found the AI prototype to introduce a clear workflow benefit by significantly shortening the time to diagnosis. This is in line with other recent studies that pointed out the potential benefits of integrating AI into radiological routine by lending the radiologist useful additional capacities to conquer workload regardless of individual experience level [31]. Our experiences while conducting the study mirrored this result; as opposed to manual segmentation, the AI prototype worked autonomously. In summary, our results imply AI-based DECT lung perfusion analysis introduce a considerably higher discriminatory power than visual assessment alone to differentiate entities at a significant workflow benefit. Especially in the setting of immunotherapy, and when considering the threat of false-negative rt-PCR test results, the implications of our study are highly relevant for clinical decision making and patient management. This study has several limitations. First, the design of this study was retrospective, and with 35 patients per group, our population was relatively small. Nonetheless, a post hoc sensitivity analysis verified the validity of our results in this setup. A prospective approach with larger sample sizes might still be helpful to confirm the implications of our results for clinical decision making. Additionally, we focused on distinguishing COVID-19 from immunotherapy-related pneumonitis, as there have been extensive reports about significant visual overlaps between these two entities. Therefore, a follow-up study to investigate the discriminative power of dual-energy CT regarding other pulmonary pathologies is merited. Moreover, image acquisition took place in a portal venous phase. As perfusion is highly susceptible to age, sex, body weight, and cardiac function, this issue can also be seen as a strength due to the compensation of possible early perfusion inhomogeneities. Nevertheless, the extracted perfusion metrics are most likely specific to our methodology, and further studies may be needed to investigate the reproducibility in other phases. Furthermore, the time to diagnosis was only measured for our senior radiologist, who is proficient with manual lung segmentation. Measuring time to diagnosis for readers without experience in manual lung segmentation might have further set manual segmentation apart from AI segmentation. Lastly, we need to address that this study was performed utilizing a high-end 3rd generation dual source scanner that is not readily available at every site. Our results might therefore be specific to this setup.

## 5. Conclusions

The investigated AI prototype positively impacts decision making and workflows by extracting perfusion metrics that differentiate COVID-19 from visually similar pneumonitis significantly faster than radiologists.

## Figures and Tables

**Table 1:** Patient characteristics.

Parameter	Female	Male	Total
Patient Population			
Absolute (*n*)	54	51	105
Reference	18	17	35
Pneumonitis	18	17	35
COVID-19	18	17	35
Mean age (y)	62 ± 13	63 ± 14	62 ± 13
Mean BMI	26 ± 1	27 ± 2	27 ± 2

**Table 2:** Number of lobe-wise CO-RADS scores for all patients.

CO-RADS Score and Level of Suspicion			Reference	Pneumonitis	COVID-19	Total (*n*)
	Level of Suspicion					
1	Very low	Normal or noninfectious	175	22	13	210
2	Low	Infectious abnormalities other than COVID-19		82	11	93
3	Indeterminate	Unclear whether COVID-19 is present		59	33	92
4	High	Infectious abnormalities suspicious for COVID-19		12	49	61
5	Very high	Infectious abnormalities typical for COVID-19			69	69

**Table 3:** Multinomial regression results, COVID-19 = reference category.

			Estimate (B)	SE	Wald *χ*^2^	*p*	Odds Ratio Exp (B)	95% CI
Differentiation from COVID-19	Pneumonitis	Reader	0.24	0.15	2.57	0.109	1.3	0.95–1.70
		CO-RADS score	−1.60	0.19	71.40	<0.001	0.20	0.14–0.29
		Iodine Uptake	0.60	0.25	5.52	0.019	1.82	1.10–2.99
		Volume	0.47	0.25	3.47	0.062	1.60	0.98–2.62
		Iodine Concentration	0.41	0.25	2.75	0.097	1.51	0.93–2.46
	Reference	Reader	0.29	0.15	3.69	0.06	1.3	0.99–1.79
		CO-RADS score	−0.11	0.02	25.10	<0.000	0.9	0.86–0.94
		Iodine Uptake	1.40	0.31	20.42	<0.000	4.07	1.03–3.42
		Volume	0.63	0.30	4.29	0.038	1.88	1.03–3.42
		Iodine Concentration	0.86	0.28	9.31	0.002	2.37	1.36–4.13

B = regression coefficient; SE = standard error, Exp (B) = Odds Ratio based on exponentiation of B, CI = Confidence Interval.

## Data Availability

Data is contained within the article.
